# Supplementary material for: Low-input proteomics identifies vWF as a negative regulator of Tet2 mutant hematopoietic stem cell expansion
Source: Cell Rep. 2025 Dec 24;45(1):116770. doi: 10.1016/j.celrep.2025.116770 (PMC12847853; doi:10.1016/j.celrep.2025.116770)
Supplement: Document S1. Figures S1–S3 [file mmc1.pdf]

Cell Reports, Volume *45*

Supplemental information

Low-input proteomics identifies vWF as a negative regulator of *Tet2* mutant hematopoietic stem cell expansion

Maria Jassinskaja, Daniel Bode, Monika Gonka, Theodoros I. Roumeliotis, Alexander J. Hogg, Juan A. Rubio Lara, Ellie Bennett, Joanna Milek, Samuel Elberfeld, Bart Theeuwes, M.S. Vijayabaskar, Lilia Cabrera Cosme, James Lok Chi Che, Sandy MacDonald, Sophia Ahmed, Benjamin A. Hall, Grace Vasey, Helena Kooi, Miriam Belmonte, Mairi S. Shepherd, William J. Brackenbury, Iwo Kucinski, Satoshi Yamazaki, Andrew N. Holding, Alyssa H. Cull, Nicola K. Wilson, Berthold Göttgens, Jyoti Choudhary, and David G. Kent

Figure S1

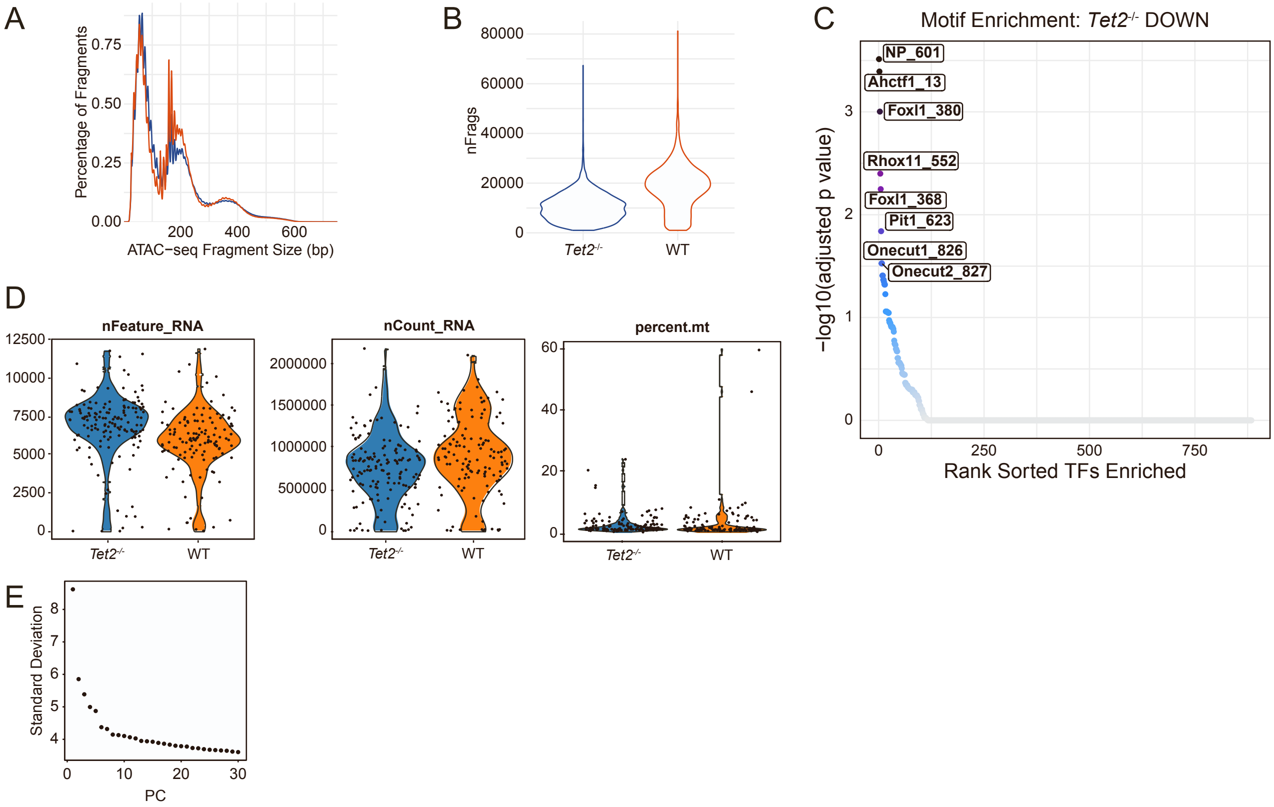
A
Percentage of Fragments
0.75
0.50
0.25
0.00
200
400
600
ATAC−seq Fragment Size (bp)
B
80000
60000
40000
20000
0
nFrags
Tet2-/-
WT
C
Motif Enrichment: Tet2-/- DOWN
NP_601
Ahctf1_13
Foxl1_380
Rhox11_552
Foxl1_368
Pit1_623
Onecut1_826
Onecut2_827
−log10(adjusted p value)
3
2
1
0
250
500
750
Rank Sorted TFs Enriched
D
nFeature_RNA
12500
10000
7500
5000
2500
0
Tet2-/-
WT
nCount_RNA
2000000
1500000
1000000
500000
0
Tet2-/-
WT
percent.mt
60
40
20
0
Tet2-/-
WT
E
Standard Deviation
8
7
6
5
4
0
10
20
30
PC

Figure S2

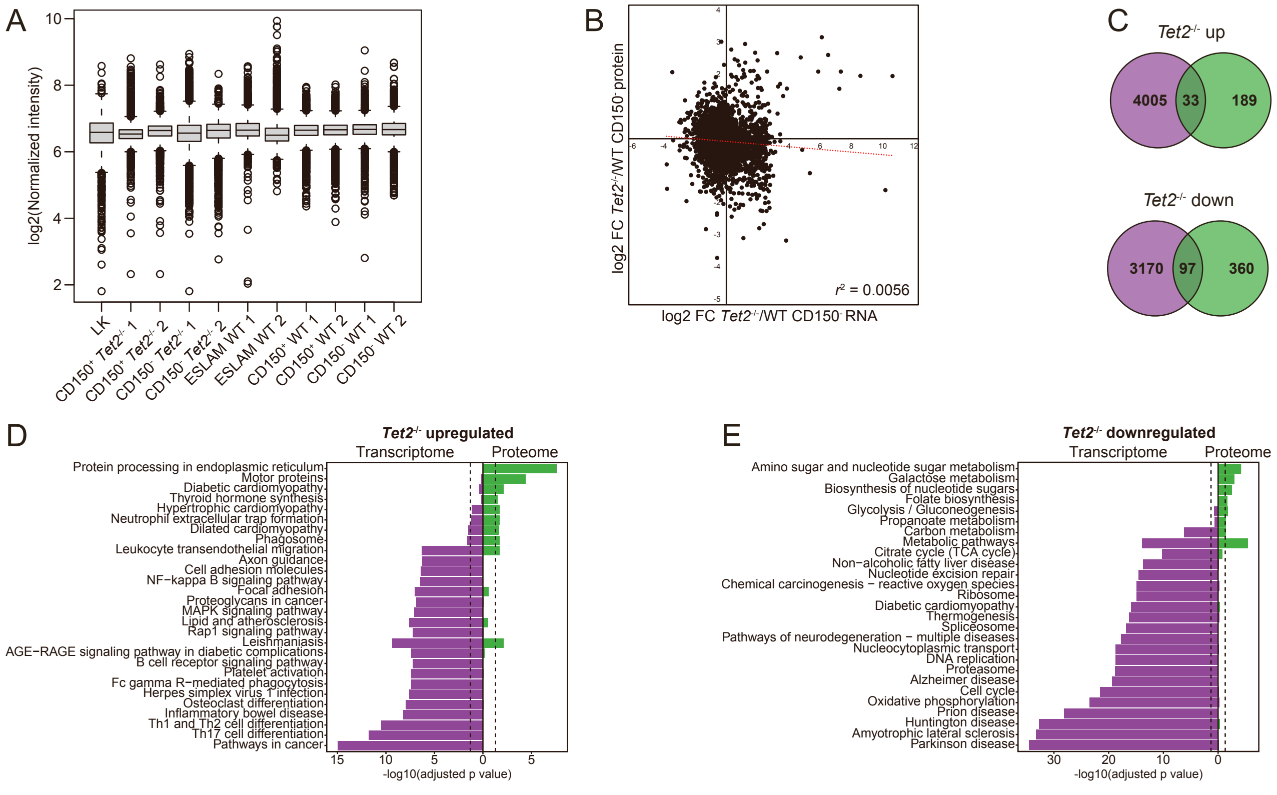

A
log2(Normalized intensity)
LK
CD150+ Tet2-/- 1
CD150+ Tet2-/- 2
CD150- Tet2-/- 1
CD150- Tet2-/- 2
ESLAM WT 1
ESLAM WT 2
CD150+ WT 1
CD150+ WT 2
CD150- WT 1
CD150- WT 2
B
log2 FC Tet2-/-/WT CD150- protein
log2 FC Tet2-/-/WT CD150- RNA
r² = 0.0056
C
Tet2-/- up
4005
33
189
Tet2-/- down
3170
97
360
D
Tet2-/- upregulated
Transcriptome
Proteome
Protein processing in endoplasmic reticulum
Motor proteins
Diabetic cardiomyopathy
Thyroid hormone synthesis
Hypertrophic cardiomyopathy
Neutrophil extracellular trap formation
Dilated cardiomyopathy
Phagosome
Leukocyte transendothelial migration
Axon guidance
Cell adhesion molecules
NF−kappa B signaling pathway
Focal adhesion
Proteoglycans in cancer
MAPK signaling pathway
Lipid and atherosclerosis
Rap1 signaling pathway
Leishmaniasis
AGE−RAGE signaling pathway in diabetic complications
B cell receptor signaling pathway
Platelet activation
Fc gamma R−mediated phagocytosis
Herpes simplex virus 1 infection
Osteoclast differentiation
Inflammatory bowel disease
Th1 and Th2 cell differentiation
Th17 cell differentiation
Pathways in cancer
-log10(adjusted p value)
E
Tet2-/- downregulated
Transcriptome
Proteome
Amino sugar and nucleotide sugar metabolism
Galactose metabolism
Biosynthesis of nucleotide sugars
Folate biosynthesis
Glycolysis / Gluconeogenesis
Propanoate metabolism
Carbon metabolism
Metabolic pathways
Citrate cycle (TCA cycle)
Non−alcoholic fatty liver disease
Nucleotide excision repair
Chemical carcinogenesis − reactive oxygen species
Ribosome
Diabetic cardiomyopathy
Thermogenesis
Spliceosome
Pathways of neurodegeneration − multiple diseases
Nucleocytoplasmic transport
DNA replication
Proteasome
Alzheimer disease
Cell cycle
Oxidative phosphorylation
Prion disease
Huntington disease
Amyotrophic lateral sclerosis
Parkinson disease
-log10(adjusted p value)

Figure S3

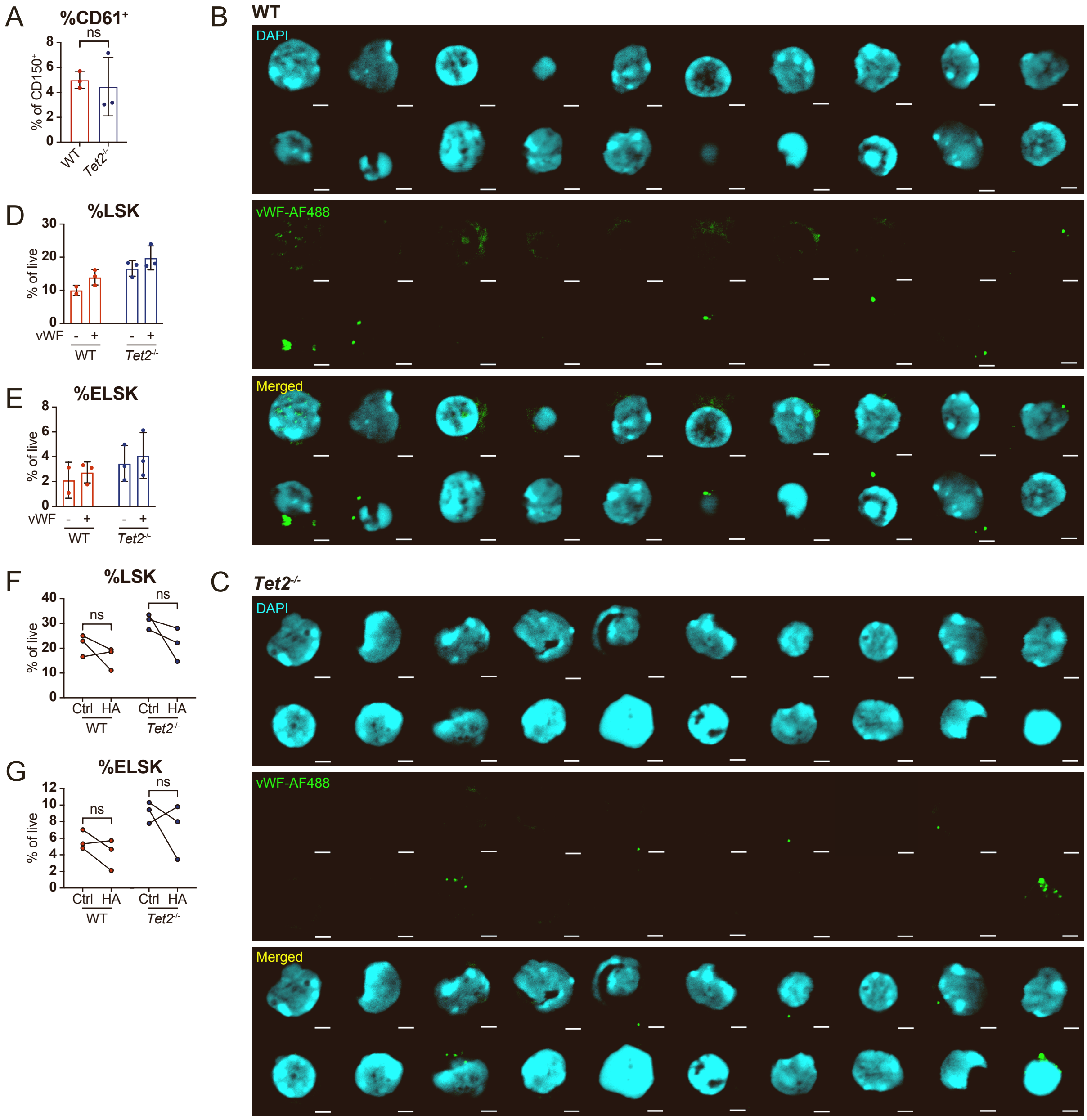

A
%CD61⁺
ns
% of CD150⁺
8
6
4
2
0
WT
Tet2-/-
B
WT
DAPI
vWF-AF488
Merged
D
%LSK
30
20
10
0
% of live
vWF
- +
- +
WT
Tet2-/-
E
%ELSK
8
6
4
2
0
% of live
vWF
- +
- +
WT
Tet2-/-
F
%LSK
40
30
20
10
0
% of live
ns
ns
Ctrl HA
Ctrl HA
WT
Tet2-/-
C
Tet2-/-
DAPI
vWF-AF488
Merged
G
%ELSK
12
10
8
6
4
2
0
% of live
ns
ns
Ctrl HA
Ctrl HA
WT
Tet2-/-

Figure S1. Quality control of scATAC-seq and scRNA-seq data. Related to Figure 1. (**A**, **B**) Fragment size distribution (**A**) and fragment distribution across samples (**B**) in scATAC-seq data. (**C**) Motif enrichment for TF motifs more accessible in WT HSC in scATAC-seq. (**D**) Number of genes detected in each cell (nFeature_RNA), total number of molecules detected within a cell (nCount_RNA), and fraction of reads mapped to mitochondrial genes (percent.mt) in scRNAs-eq data. (**E**) Elbow plot for scRNAseq data. The top 5 PCs were selected for the downstream analysis.

Figure S2. Proteome of CD150^{-} short term (ST)-HSCs undergoes extensive remodeling upon loss of TET2. Related to Figure 3. (**A**) Normalized intensity across *Tet2*$^{-/-}$ and WT HSPC samples analyzed by LC-MS/MS. (**B**) Correlation between protein and gene expression differences (log2 FC) between *Tet2*$^{-/-}$ and WT CD150^{-} cells. The dotted red line indicates the linear trendline. r^2 represents the Pearson correlation coefficient. (**C**) Overlap between candidate target genes and proteins enriched or depleted in *Tet2*$^{-/-}$ relative to WT CD150^{-} cells. (**D**, **E**) KEGG pathway analysis of candidate target proteins and genes enriched (**D**) or depleted (**E**) in *Tet2*$^{-/-}$ relative to WT CD150^{-} cells. The dotted lines mark adjusted p-value = 0.05.

Figure S3. Impact of vWF and hyaluronan on expansion of *Tet2*$^{-/-}$ HSCs in culture. Related to Figure 5. (**A**) Frequency of ITGB3/CD61^{+} cells within WT and *Tet2*$^{-/-}$ CD150^{+} cells. N = 3 individual mice per genotype. (**B**, **C**) Immunofluorescence images of vWF in WT (**B**) and *Tet2*$^{-/-}$ (**C**) CD150^{+} cells. Scale bar represents 2 μM. (**D**, **E**) Frequency of HSPCs (LSK; **D**) and HSCs (ELSK; **E**) following 28-day culture of WT and *Tet2*$^{-/-}$ HSCs on tissue-culture plates functionalized with vWF. N = 2-3 individual mice per genotype. (**F**, **G**) Frequency of HSPCs (LSK; **F**) and HSCs (ELSK; **G**) following 28-day culture of WT and *Tet2*$^{-/-}$ HSCs on hydrogels functionalized with hyaluronan (HA). N = 3 individual mice per genotype. ns = non-significant.

Table S1

Click here to access/download
Supplemental Videos and Spreadsheets
Table_S1_scATACseq.xlsx

Table S2

Click here to access/download
Supplemental Videos and Spreadsheets
Table_S2_revised.xlsx

Table S3

Click here to access/download
Supplemental Videos and Spreadsheets
Table_S3_HoxB8_proteome.xlsx

Table S4

Click here to access/download
Supplemental Videos and Spreadsheets
Table_S4_HSC_proteome.xlsx
